# Supplementary material for: 3D genome of multiple myeloma reveals spatial genome disorganization associated with copy number variations
Source: Nat Commun. 2017 Dec 5;8:1937. doi: 10.1038/s41467-017-01793-w (PMC5715138; doi:10.1038/s41467-017-01793-w)
Supplement: Supplementary file 1 — Supplementary Information [file 41467_2017_1793_MOESM1_ESM.pdf]

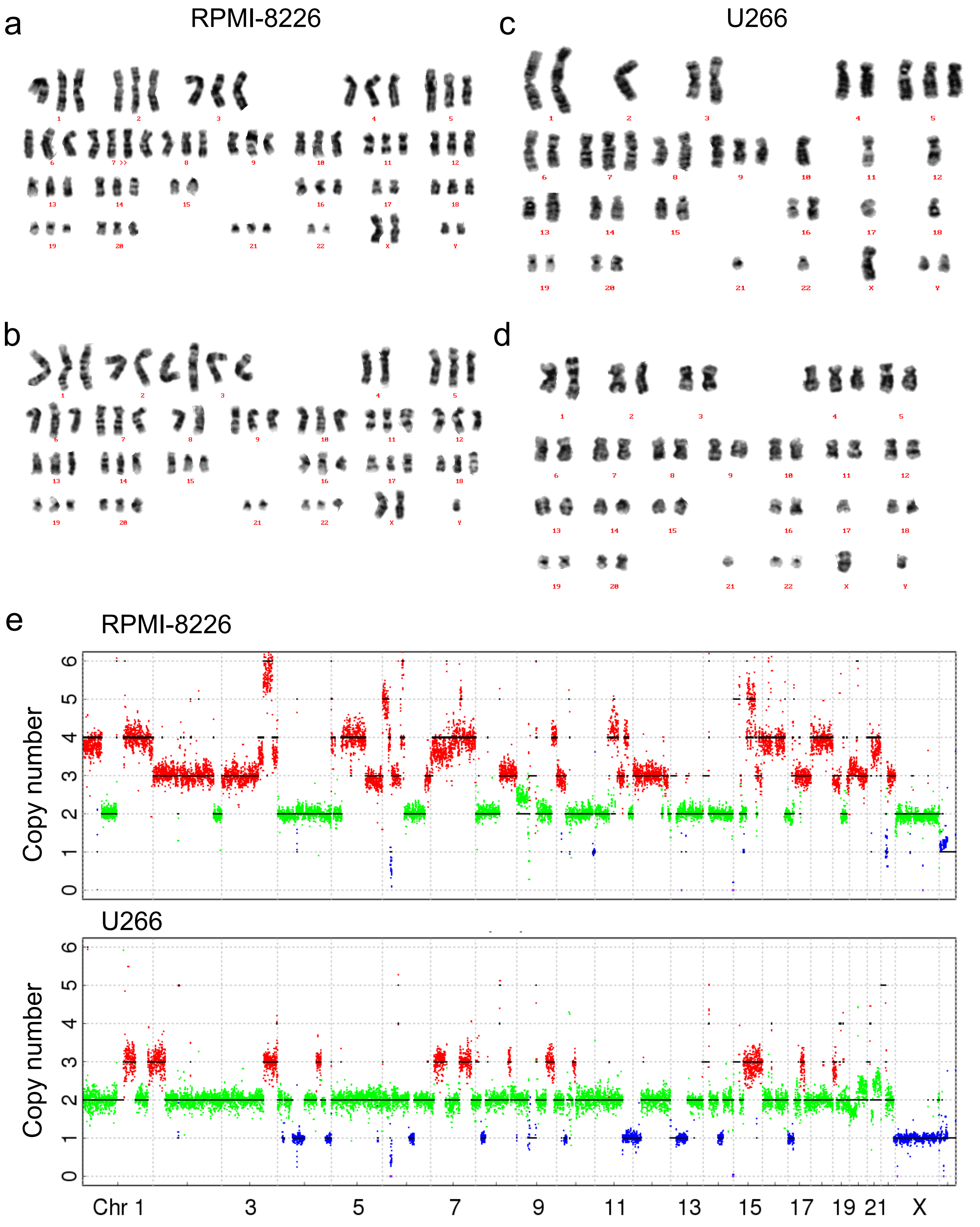

Supplementary Figure 1. Karyotypes of RPMI-8226 and U266 cells. (**a**–**b**) Karyotypes of RPMI-8226 cell line. (**c**–**d**) Karyotypes of U266 cell line. (**e**) Estimated CNVs in RPMI-8226 and U266 using WGS data.

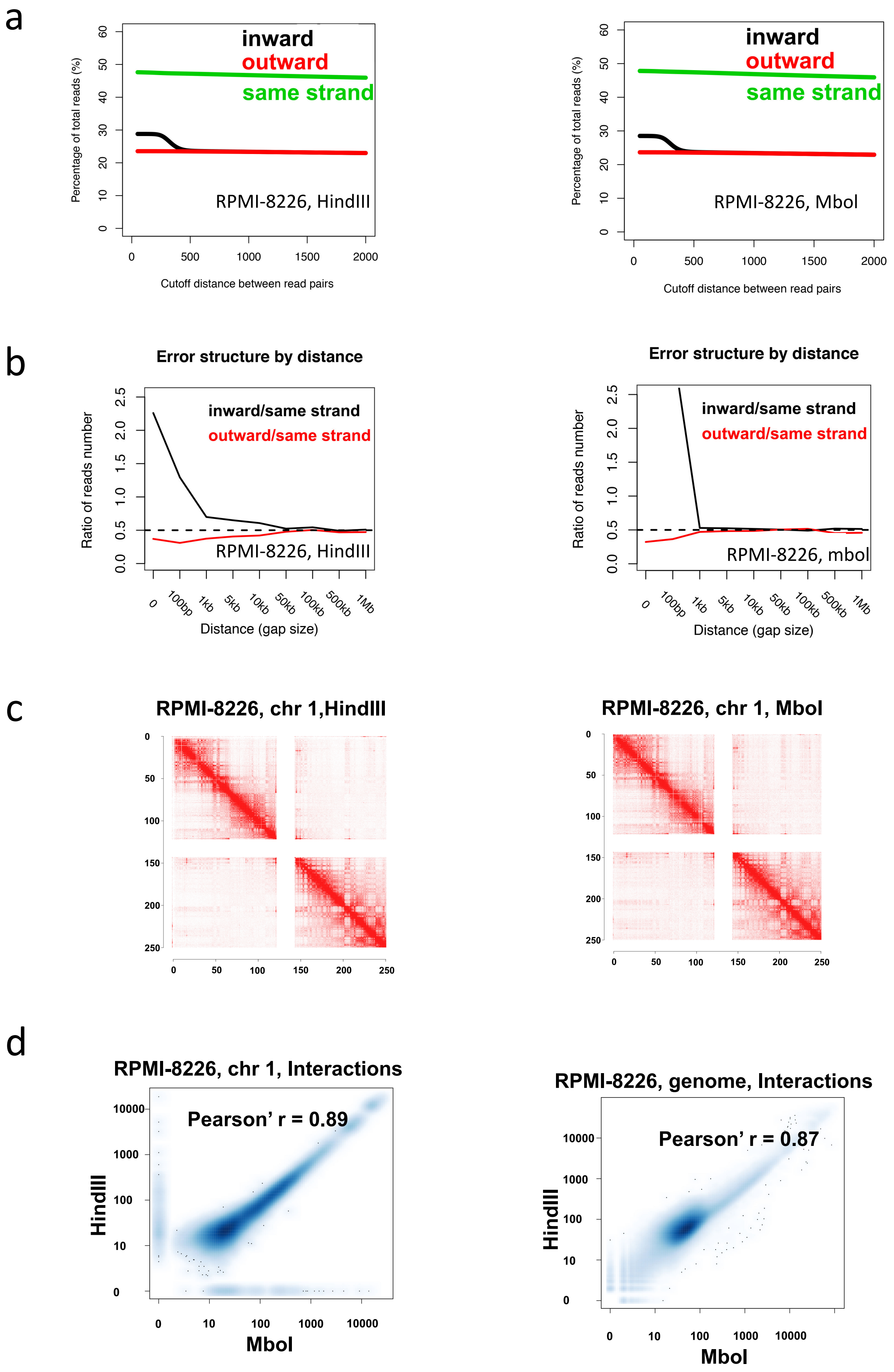

a
inward
outward
same strand
Percentage of total reads (%)
RPMI-8226, HindIII
Cutoff distance between read pairs
RPMI-8226, MboI
b
Error structure by distance
inward/same strand
outward/same strand
Ratio of reads number
RPMI-8226, HindIII
RPMI-8226, mboI
Distance (gap size)
0
100bp
1kb
5kb
10kb
50kb
100kb
500kb
1Mb
c
RPMI-8226, chr 1,HindIII
RPMI-8226, chr 1, MboI
d
RPMI-8226, chr 1, Interactions
Pearson' r = 0.89
RPMI-8226, genome, Interactions
Pearson' r = 0.87
HindIII
MboI

Supplementary Figure 2. Basic characterization of Hi-C, WGS and RNA-seq data. (**a**) Relative composition of different classes of Hi-C reads at different ranges of genomic spans. **(b)** Composition of paired-end Hi-C reads in different Hi-C experiments, as a function of the genomic distance between a pair of DNA fragments. **(c)** Contact matrix from the whole chromosome 1 at 200 kb resolution. Left: RPMI-8226 with HindIII, right: RPMI-8226 with Mbo I. (d) Left: Comparing interaction scores of chromosome 1 in RPMI-8226 (HindIII) at y-axis and RPMI-8226 (MboI) at x-axis, 200 kb resolution, the Pearson's r is 0.89; Right: Comparing interaction scores of whole genome in RPMI-8226 (HindIII) at y-axis and RPMI-8226 (MboI) at x-axis, at 3 M resolution, the Pearson's r is 0.87.

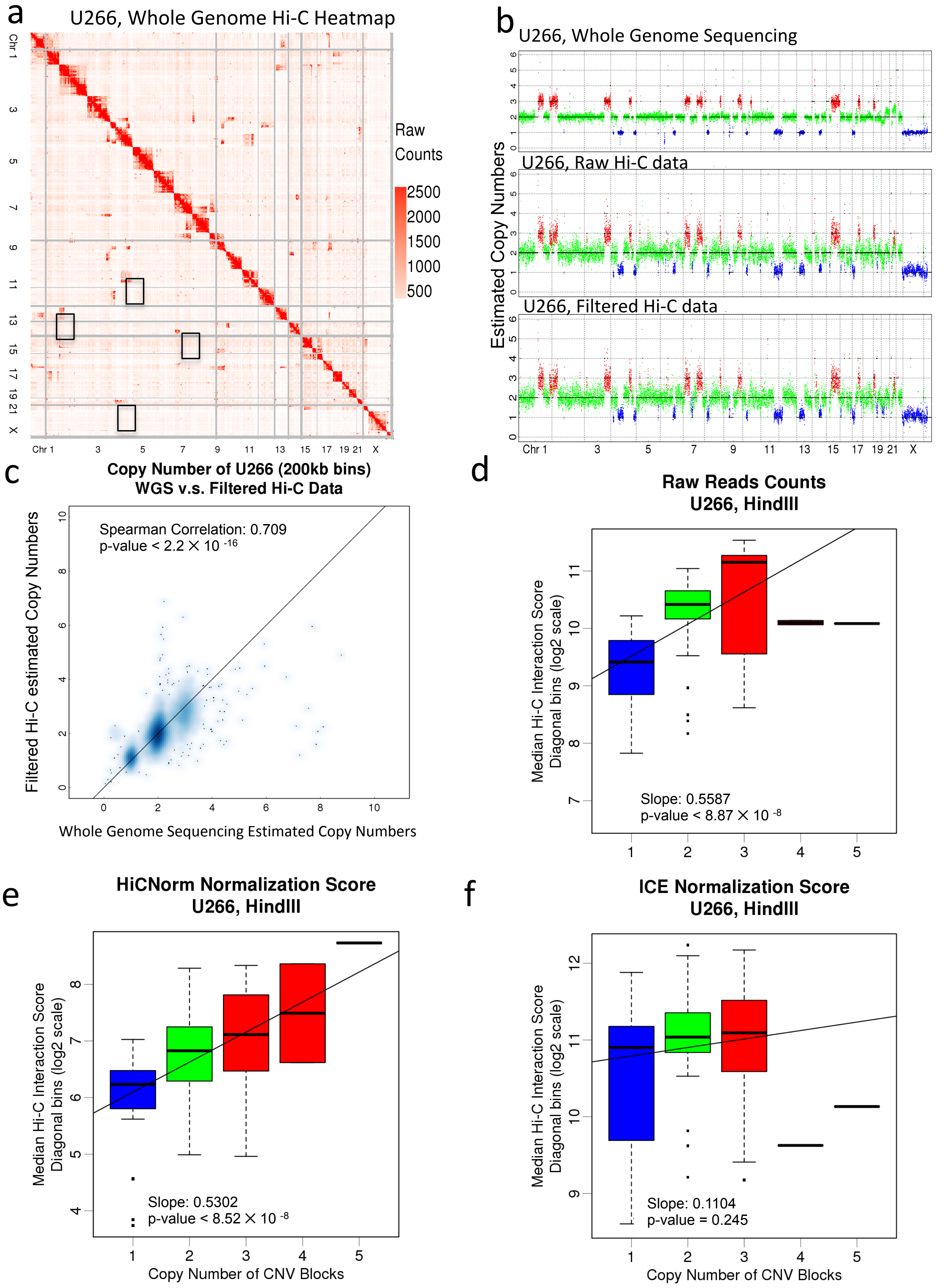

Supplementary Figure 3. Correcting copy number variation in cancer Hi-C data of U266 cells. See Figure 1 legend. The data are for U266 cells.

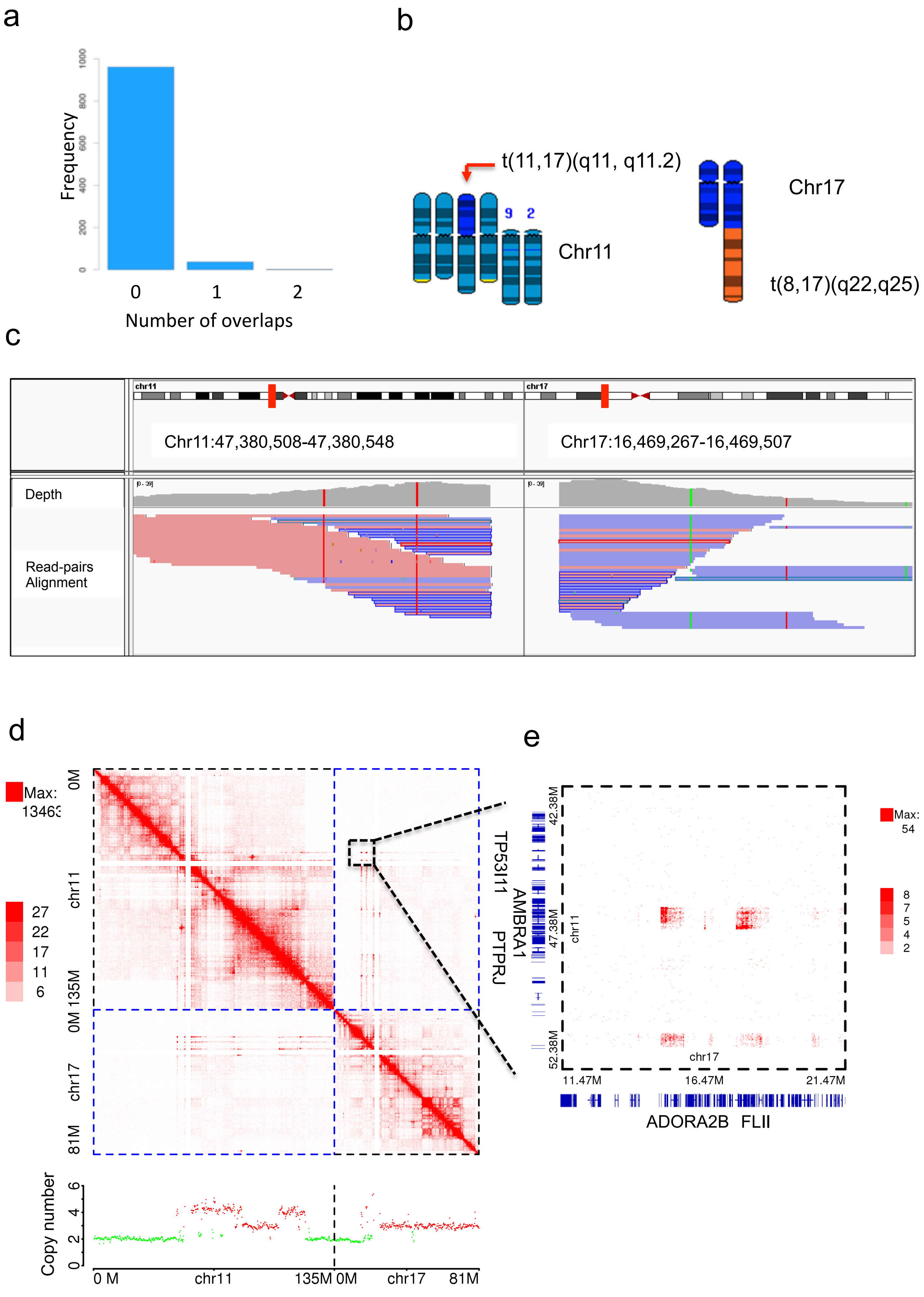
a
Frequency
Number of overlaps
b
t(11,17)(q11, q11.2)
Chr17
Chr11
t(8,17)(q22,q25)
c
Chr11:47,380,508-47,380,548
Chr17:16,469,267-16,469,507
Depth
Read-pairs Alignment
d
Max:
27
22
17
11
6
chr11
chr17
TP53I11
AMBRA1
PTPRJ
Copy number
e
Max:
54
ADORA2B
FLII

Supplementary Figure 4. In RPMI-8226, both Hi-C and WGS data identified the translocation of t(11,17)(q11, q11.2). (a) Overlap between random sites and translocation sites in RPMI 8226 cells. 100 random-sampled sites were used to calculated the overlap with WGS translocations for 1000 times. (b) The chimeric chromosome formed between chr11 and chr17 is identified by spectral karyotyping (SKY). The SKY image of RPMI-8226 is from the NCBI SKY database. (c) The translocation event of t(11;17)(q11;q11.2) in RPMI-8226 cells plotted by IGV tool, with only WGS paired reads supporting this translocation event. The reads depth and alignment panels are shown. Read alignments are sorted by read location and colored by read strands. (d) Hi-C interaction maps of chromsome 11 and 17 showed the translocation of t(11;17)(q11;q11.2) in RPMI-8226. Top panel: the intra and inter-chromosome interaction maps of chromosome 11 and 17 in RPMI-8226. Bottom panel: copy number variations of chromosome 11 and 17. (e) Enlarged inter-chromosomal interactions induced by the translocation event. Genes of ADOR2ZB, FLII, AMBRA1 and PTRPJ were affected by inter-chromosomal translocations. The AMBRA1 gene is a member of autophagy signaling network and recently been identified as a haploinsufficient tumor suppressor gene by regulating c-Myc[1]. ADORA2B is a member of the G protein-coupled receptor superfamily and involved in the metastasis of breast cancer (PMC3612632).

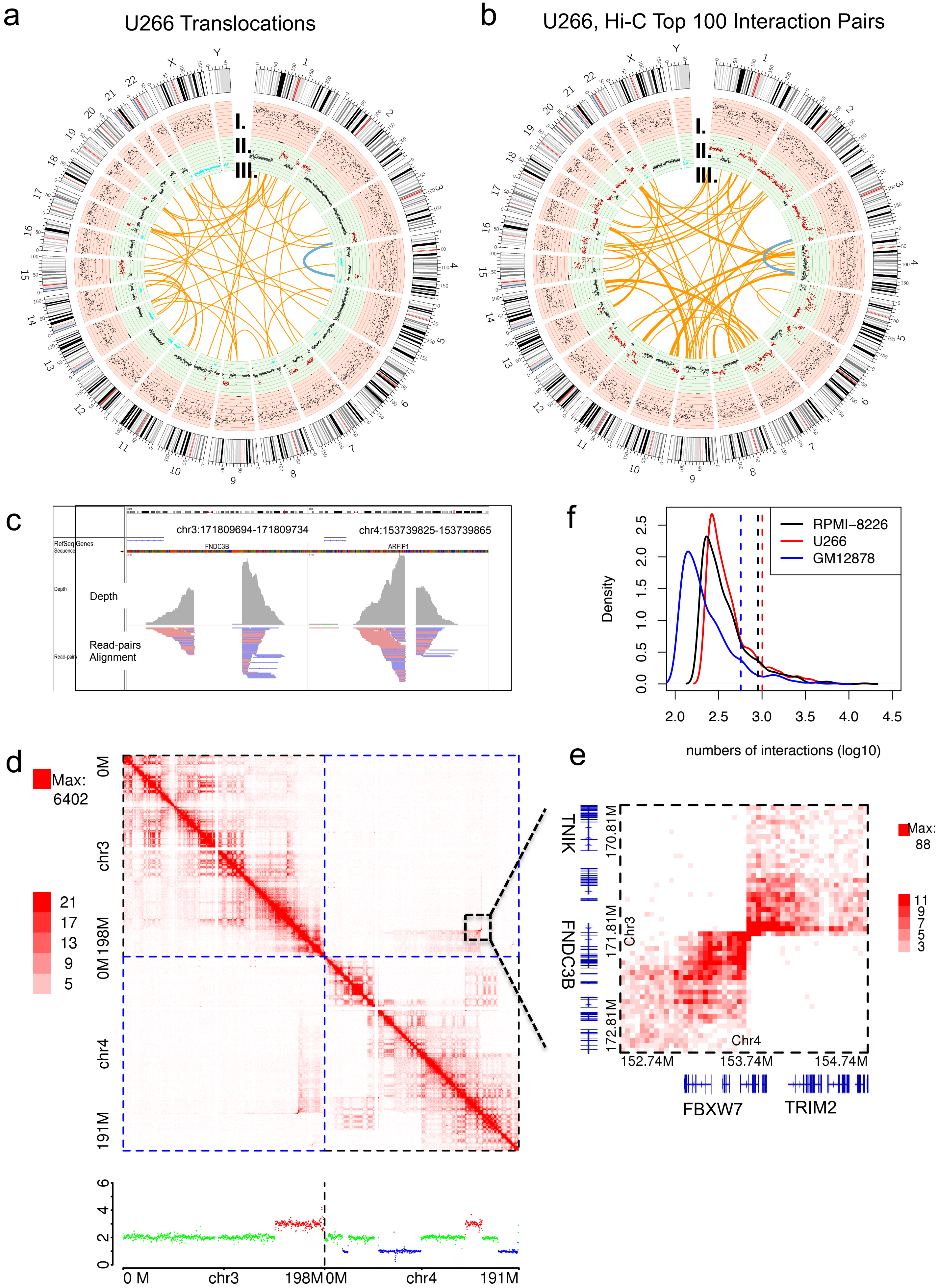

a
U266 Translocations
b
U266, Hi-C Top 100 Interaction Pairs
I.
II.
III.
c
chr3:171809694-171809734
chr4:153739825-153739865
RefSeq Genes
Sequence
FNDC3B
ARFIP1
Depth
Read-pairs Alignment
f
RPMI−8226
U266
GM12878
Density
numbers of interactions (log10)
d
Max: 6402
21
17
13
9
5
0M
chr3
198M
0M
chr4
191M
0 M
chr3
198M
0M
chr4
191M
e
TNIK
FNDC3B
170.81M
171.81M
172.81M
Chr3
Chr4
152.74M
153.74M
154.74M
FBXW7
TRIM2
Max: 88
11
9
7
5
3

Supplementary Figure 5. In U266 cells, both Hi-C and WGS data identified the translocation between gene FNDC3B at 3q26.31 and gene FBXW7 at 4q33.2. (a)(b) See Figure 2 (a)(b) legends, The 42 inter-chromosome translocation events and top 100 highest inter-chromosome interactions, RNA-seq counts and copy numbers of U266 cells are used for circos plot . (c) The translocation event of t(3, 4)(q26.31, q31.3) in U266 cells plotted by IGV tool, with only WGS paired reads supporting this translocation event. The reads depth and alignment panels are shown. Read alignments are sorted by read location and colored by read strands. (d) Hi-C interaction maps of chromsome 3 and 4 showed the translocation of t(3, 4)(q26.31, q31.3) in U266. Top panel: the intra and inter-chromosome interaction maps of chromosome 3 and 4 in U266. Lower panel: the copy number variations of chromosome 3 and 4. (e) Enlarged inter-chromosomal interactions induced by the translocation event t(3, 4)(q26.31, q31.3) in U266. Genes of FNDC3B, FBXW7 and TRIM2 were affected by inter-chromosomal translocations. FNDC3B is a recently identified oncogene amplified in hepatocellular carcinoma and induces epithelial-to-mesenchymal transition [2]. FBXW7 is a ubiquitin ligase and tumor suppressor in many cancer types [3]. In MM, FBXW7 is a pro-survival factor through degradation of p100 which target on NF-κB signaling [4]. (f) Density plot for top 1000 inter-chromosomal interactions in three cell lines. Dash lines indicate 10% cutoff.

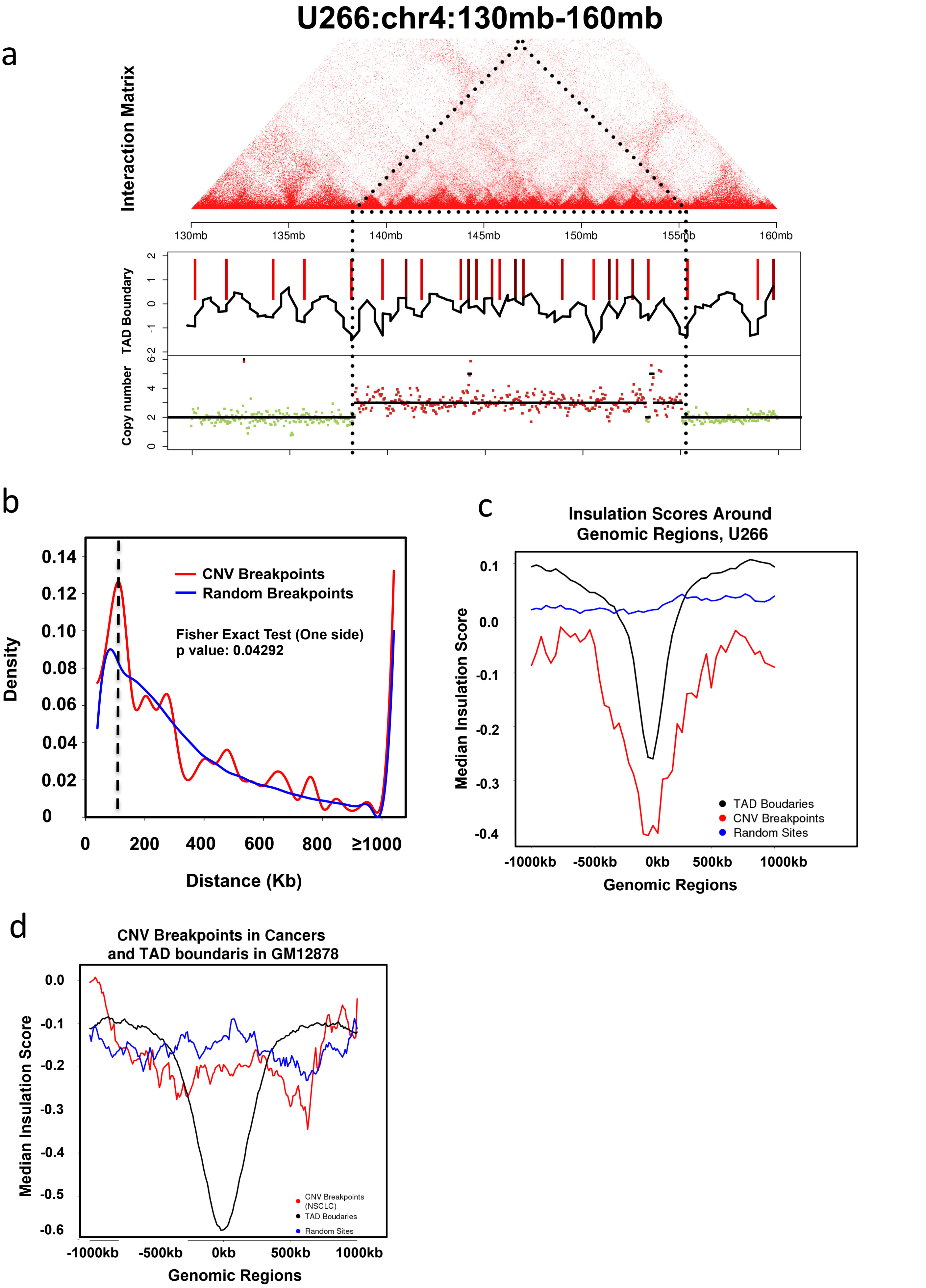

Supplementary Figure 6. CNV breakpoints associated with TAD boundaries in U266 cells. See Figure 3 legends. The data are for U266 cells.

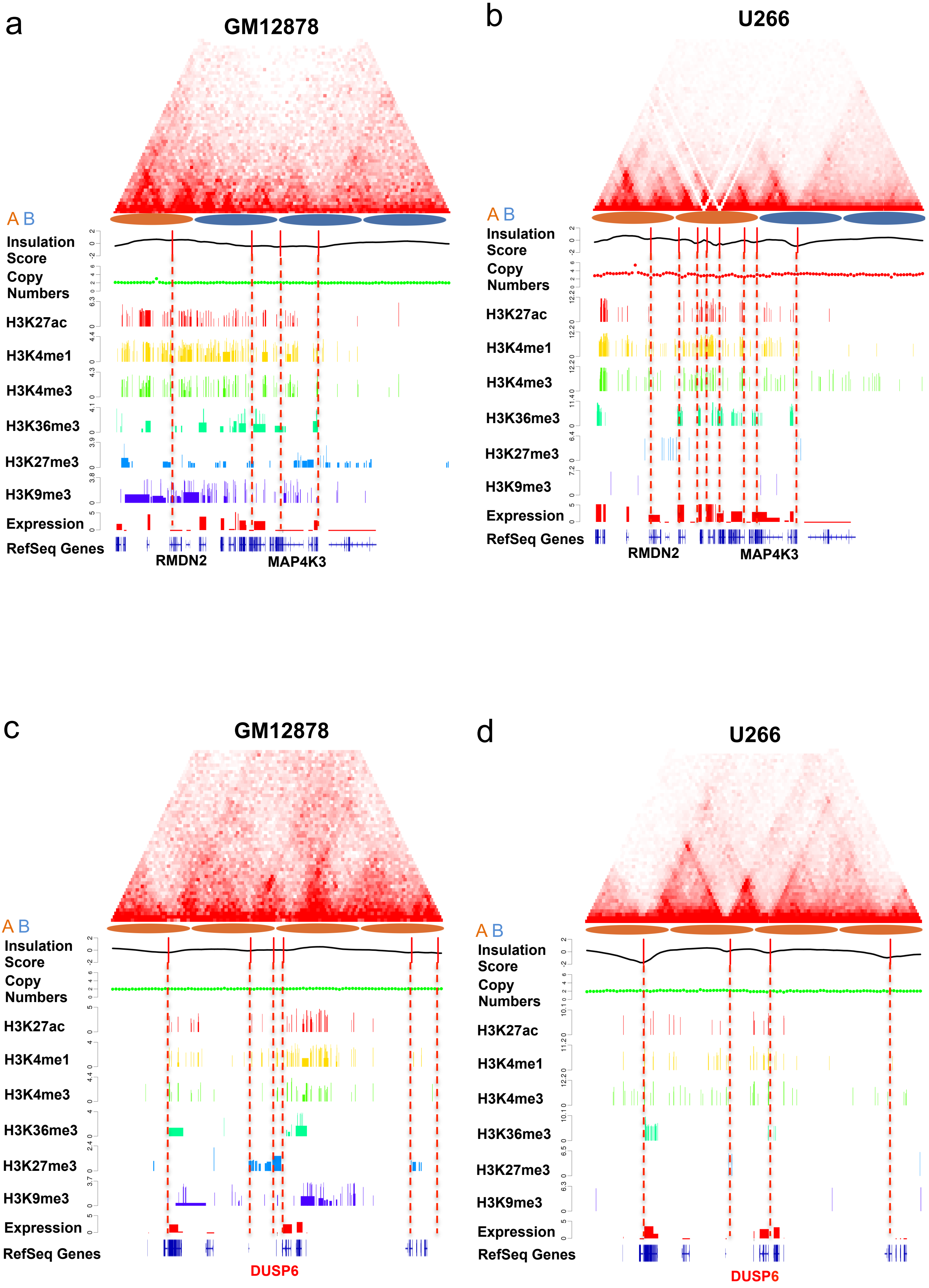
a
GM12878
A B
Insulation Score
Copy Numbers
H3K27ac
H3K4me1
H3K4me3
H3K36me3
H3K27me3
H3K9me3
Expression
RefSeq Genes
RMDN2
MAP4K3
b
U266
A B
Insulation Score
Copy Numbers
H3K27ac
H3K4me1
H3K4me3
H3K36me3
H3K27me3
H3K9me3
Expression
RefSeq Genes
RMDN2
MAP4K3
c
GM12878
A B
Insulation Score
Copy Numbers
H3K27ac
H3K4me1
H3K4me3
H3K36me3
H3K27me3
H3K9me3
Expression
RefSeq Genes
DUSP6
d
U266
A B
Insulation Score
Copy Numbers
H3K27ac
H3K4me1
H3K4me3
H3K36me3
H3K27me3
H3K9me3
Expression
RefSeq Genes
DUSP6

Supplementary Figure 7. Example of MAPK pathway gene expression changes associated with spatial genome reorganization. (a) Expression and Hi-C heatmap of gene MAP4K3 in GM12878. (b) Expression and Hi-C heatmap of gene MAP4K3 in U266. Up-regulation of MAP4K3 is associated with both copy number gain and spatial genome reorganization. (c) Expression and Hi-C heatmap of gene DUSP6 in GM12878. (d) Expression and Hi-C heatmap of gene DUSP6 in U266. Up-regulation of DUSP6 is associated with spatial genome reorganization but not copy number variation.

Supplementary Table 1. Summary of sequencing data.

	Cell type	Enzyme	All reads	Filtered reads	Cis ratio
Hi-C	RPMI-8226	HindIII	217,829,046	92,668,774 (42.5%)	74.30%
	RPMI-8226	MboI	122,227,090	81,041,306 (66.3%)	60.50%
	U266	HindIII	228,423,540	86,378,574 (37.8%)	77.10%
	U266	MboI	207,191,357	131,383,082 (63.4%)	64.77%
DNA-seq (WGS)	RPMI-8226	-	619,194,153	-	-
	U266	-	646,773,193	-	-
RNA-seq	RPMI-8226	-	55,945,480 (3 repeats)	-	-
	U266	-	57,424,004 (3 repeats)	-	-

Supplementary References

1. Cianfanelli, V. *et al.* AMBRA1 links autophagy to cell proliferation and tumorigenesis by promoting c-Myc dephosphorylation and degradation. *Nat Cell Biol* **17**, 20-30 (2015).
2. Cai, C. *et al.* Activation of multiple cancer pathways and tumor maintenance function of the 3q amplified oncogene FNDC3B. *Cell cycle* **11**, 1773-1781 (2012).
3. Yumimoto, K. & Nakayama, K.I. Fbxw7 suppresses cancer metastasis by inhibiting niche formation. *Oncoimmunology* **4**, e1022308 (2015).
4. Busino, L. *et al.* Fbxw7alpha- and GSK3-mediated degradation of p100 is a pro-survival mechanism in multiple myeloma. *Nature cell biology* **14**, 375-385 (2012).
