# Supplementary material for: 3D genome of multiple myeloma reveals spatial genome disorganization associated with copy number variations
Source: Nat Commun. 2017 Dec 5;8:1937. doi: 10.1038/s41467-017-01793-w (PMC5715138; doi:10.1038/s41467-017-01793-w)
Supplement: Supplementary file 3 — Description of Additional Supplementary Files [file 41467_2017_1793_MOESM3_ESM.pdf]

Description of Additional Supplementary Files

File Name: Supplementary Data 1
Description: Translocation Events.

File Name: Supplementary Data 2
Description: Gene lists associated with translocation events.
